# Analysis of the nutritional profile after endoscopic gastroplasty in the treatment of obesity

**DOI:** 10.1590/0102-672020260000042e1971

**Published:** 2026-09-28

**Authors:** Adonay Ferreira LISBOA, Marcelo Falcão de SANTANA

**Affiliations:** 1 Escola Bahiana de Medicina e Saúde Pública, Faculty of Medicine – Salvador (BA), Brazil.; 2 Falcão Institute of Endoscopy and Surgery – Salvador (BA), Brazil.

**Keywords:** Obesity, Gastroplasty, Endoscopy, Nutritional Status, Malnutrition, Obesidade, Gastroplastia, Endoscopia, Estado Nutricional, Desnutrição

## Abstract

**Background:**

Endoscopic gastroplasty (EG) has emerged as a promising technique for the treatment of obesity. However, data on the nutritional profile of patients after this surgical procedure are scarce in the literature.

**Aims:**

To analyze the nutritional profile of patients who underwent EG for the treatment of obesity during the postoperative period.

**Methods:**

This retrospective observational study investigated medical record data from a private healthcare service in Salvador, Bahia, where EG was performed EG. Epidemiological and laboratory data (total protein, albumin, calcium, zinc, vitamin B12, 25-hydroxyvitamin D, hemoglobin, glucose, glycated hemoglobin, ferritin, and iron) were collected before and after surgery over a 6-month period. A p-value>0.05 was considered statistically significant.

**Results:**

Twenty-seven patients were analyzed, with a mean age of 43.8 (±13.3) years, of whom 88.9% were female. The patients’ initial body mass index (BMI) was 35.31±8.7 kg/m^2^. Total weight loss was 10±5.7, and excess weight loss was 41.1±28.1. Preoperatively, iron deficiency was observed in 1 patient, vitamin B12 deficiency in 2 patients, 25-hydroxyvitamin D deficiency in 2 patients, zinc deficiency in 1 patient, calcium deficiency in 2 patients, and hyperglycemia in 4 patients. No consequences of these deficiencies were observed during follow-up. There was no correlation between BMI and laboratory data (p>0.05).

**Conclusion:**

The nutritional profile remained stable before and after EG, with sustained weight loss.

## ARTICLE HIGHLIGHTS

No significant changes in nutritional laboratory parameters were observed after 6 months of follow-up.

EG promoted sustained weight loss during follow-up period.

In this sample, body mass index showed no correlation with nutritional laboratory markers.

## Summary

**CENTRAL MESSAGE:**

The surgical treatment of obesity currently involves laparoscopic surgery and bariatric therapeutic endoscopy, with technical variations that are increasingly adapted to individual patient circumstances. The most common laparoscopic options performed are sleeve gastrectomy (SG), Roux-en-Y gastric bypass (RYGB), and duodenal switch, with other emerging laparoscopic surgical techniques also aiming to induce malabsorption through manipulation of the small intestine. In primary obesity therapy, intragastric balloon implantation and endoscopic gastroplasty (EG) are minimally invasive endoscopic procedures. EG involves performing sutures on the anterior and posterior walls of the gastric body, creating a tubular configuration defined by the lesser curvature and angular incisure, as the greater curvature of the stomach is reduced.

**PERSPECTIVES:**

These findings show that the nutritional profile remained stable before and after EG in the short term (up to 6 months). Sustained weight loss was observed throughout the follow-up period, and body mass index and laboratory data before the procedure were not correlated. However, due to the reduced statistical power of this sample and the incompleteness of the data, the implications of EG for potential nutritional deficiencies in the management of obesity remain uncertain.

## VISUAL ABSTRACT

**Figure:**
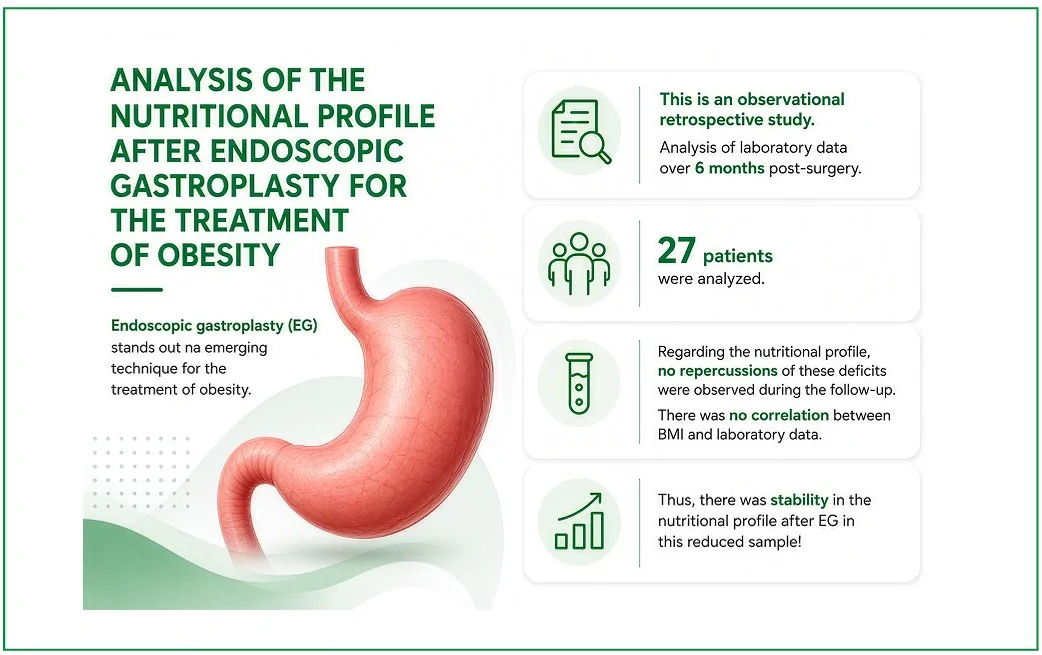

## RESUMO

**Racional:**

A gastroplastia endoscópica (GE) destaca-se como uma técnica emergente para o tratamento da obesidade. No entanto, dados sobre o perfil nutricional de pacientes após esse procedimento cirúrgico são escassos na literatura.

**Objetivos:**

Analisar o perfil nutricional de pacientes submetidos à gastroplastia endoscópica para o tratamento da obesidade no período pós-operatório.

**Métodos:**

Estudo observacional retrospectivo que investigou dados de prontuários médicos de um serviço privado em Salvador/BA que realizou a gastroplastia endoscópica. Dados epidemiológicos e laboratoriais (proteína total, albumina, cálcio, zinco, vitamina B12, 25-hidroxivitamina D, hemoglobina, glicose, hemoglobina glicada, ferritina e ferro) foram coletados antes e após a cirurgia, durante o período de 6 meses. Um valor de p>0,05 foi considerado estatisticamente significativo.

**Resultados:**

Vinte e sete pacientes foram analisados, com idade média de 43,8 (±13,3) anos, dos quais 88,9% eram do sexo feminino. O IMC inicial dos pacientes foi de 35,31±8,7 kg/m^2^. A %TWL foi de 10±5,7 e a %EWL foi de 41,1±28,1. No pré-operatório, observou-se deficiência de ferro em 1 paciente, de vitamina B12 em 2 pacientes, de 25-hidroxivitamina D em 2 pacientes, de zinco em 1 paciente, de cálcio em 2 pacientes e hiperglicemia em 4 pacientes. Não foram observadas repercussões dessas deficiências durante o acompanhamento. Não houve correlação entre o IMC e os dados laboratoriais (p>0,05).

**Conclusões:**

Houve estabilidade no perfil nutricional antes e depois da gastroplastia endoscópica, com perda de peso sustentada.

## INTRODUCTION

 Obesity is a chronic metabolic disorder that alters endocrine, cardiovascular, and pulmonary functions^25^ . This disease is closely related to the risk of venous thromboembolism, stroke, hypertension, acute myocardial infarction, type II diabetes mellitus (DM2), metabolic dysfunction-associated steatotic liver disease, atherosclerosis, arrhythmias, obstructive sleep apnea, among other conditions associated with morbidity and mortality^9,17,22,24,26^ . 

 The surgical treatment of obesity currently involves laparoscopic surgery and bariatric therapeutic endoscopy, with technical variations that are increasingly adapted to individual patient circumstances. The most common laparoscopic options performed are sleeve gastrectomy (SG), Roux-en-Y gastric bypass (RYGB), and duodenal switch, with other emerging laparoscopic surgical techniques also aiming to induce malabsorption through manipulation of the small intestine^2,17,19,21^. In primary obesity therapy, intragastric balloon implantation and endoscopic gastroplasty (EG) are minimally invasive endoscopic procedures^10^ . 

 EG using the Apollo method was developed in the USA in the 1990s, achieving good results in weight loss and control of DM2 and other related comorbidities. EG involves performing sutures with Fibloc Permanent^®^ 2.0 sutures (monodirectional smooth suture thread) on the anterior and posterior walls of the gastric body, creating a tubular configuration defined by the lesser curvature and angular incisure, as the greater curvature of the stomach is reduced. In this way, restriction of the tubularized gastric volume leaves the patient satiated after ingesting small amounts of food, mimicking the tubular appearance acquired with the sleeve^3, 11^. 

 This procedure, as well as other surgical interventions, allows for weight loss and better control of obesity-related comorbidities in a safe and effective manner^3,14,19^. However, caution is needed regarding the patient’s nutritional pattern, since a diet poor in micronutrients and rich in carbohydrates and fats, which is already common among patients with obesity, can contribute to nutritional deficiencies^20,25^. Therefore, according to the American Society for Metabolic and Bariatric Surgery (ASMBS), monitoring nutritional status by assessing vitamins B12, A, E, K, and D, as well as folic acid, iron, and calcium, is fundamental to dietary management^5^ . 

 On the other hand, there is a lack of robust data establishing a correlation between nutritional deficiencies and patients in the postoperative period following EG in the short and long term. 

 This study aimed to analyze the nutritional profile of patients undergoing EG for the treatment of obesity. 

## METHODS

 This retrospective observational cohort study was conducted in Salvador, Bahia (BA), at the Falcão Institute of Endoscopy and Surgery (*Instituto Falcão de Endoscopia e Cirurgia* — IFEC). The study was conducted in accordance with the National Health Council (*Conselho nacional de Saúde* — CNS) Resolution 466/12 and was approved by the Research Ethics Committee of the Bahiana School of Medicine and Public Health (CAAE 85931025.8.0000.5544). 

### Study population

 All study participants underwent EG performed by a single endoscopist and bariatric surgeon. All patients were evaluated and followed by a single psychologist and a single nutritionist. 

### Inclusion criteria

 Patients who underwent EG between January 2017 and December 2023 and were followed up at the private clinic in Salvador, BA, were included. 

### Exclusion criteria

 Patients under 18 years of age, those who had undergone other gastric procedures, such as intragastric balloon placement or bariatric surgery, and those who used iron supplements were excluded. 

### Sample size

 The sample was selected by convenience and included all patients who underwent EG, totaling 96 individuals (Figure 1). 

**Figure 1:**
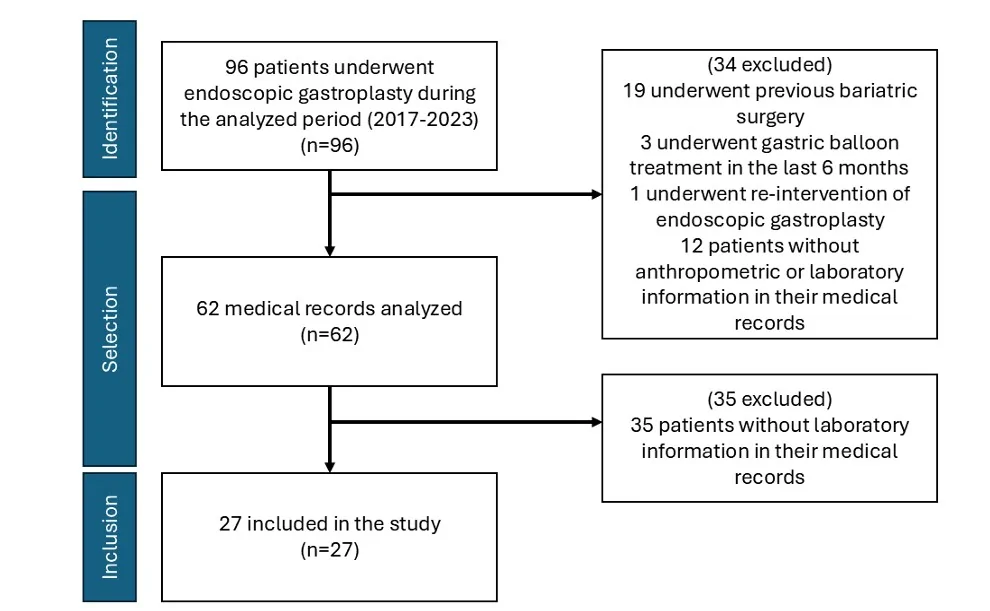
Patient characteristics.

### Endoscopic gastroplasty

 The patient, under general anesthesia, is positioned in the left lateral decubitus position. Upper digestive endoscopy is performed orally with an OLYMPUS CV-150 2T and CV170 endoscope. The EG procedure is performed according to the Apollo method of the OverStitch^®^ suture system (Apollo Endosurgery, Austin, TX, USA), as shown in Figure 2. Gastric insufflation for the endoscopic procedure is performed with CO2. Immediately afterward, 3–5 intraluminal sutures are made in a hybrid pattern, with linear stitches in the first row and a “W” stitch (Figure 3) in the return suture line of the “U,” along the entire greater curvature, using Filbloc Permanent ^®^ 2-0 (smooth omnidirectional suture thread) in a transluminal manner, creating tubularization of the gastric chamber from the lesser curvature to the esophagogastric junction. These sutures begin at the angular incisure and extend to the gastric body, sparing the fundus. The order is as follows: the anterior wall, passing through the greater curvature, and then the posterior wall of the stomach, with the appearance of the procedure before and after suturing shown in Figure 4. Afterwards, the result of the procedure is reviewed and washed with saline solution, with aspiration of the liquid content and clots. The procedure lasts approximately 50 minutes. 

**Figure 2:**
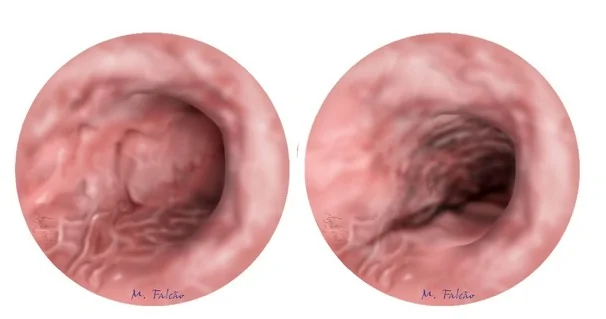
Comparison of the luminal endoscopic appearance of the stomach before (left) and after (right) endoscopic gastroplasty (insufflated with CO _2_).

**Figure 3:**
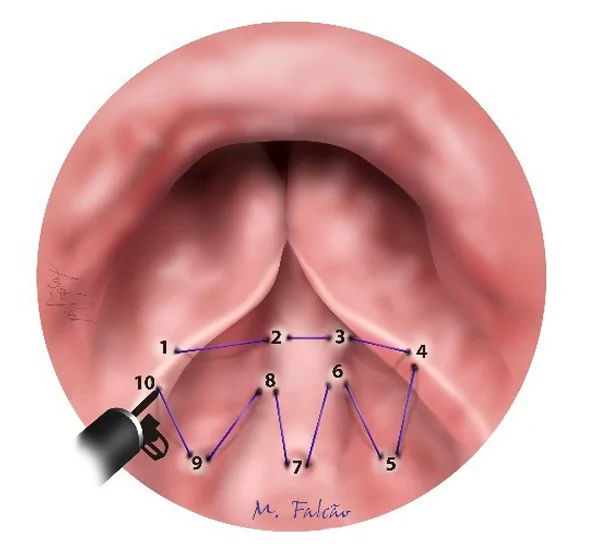
W-shaped suture pattern performed during endoscopic gastroplasty.

**Figure 4:**
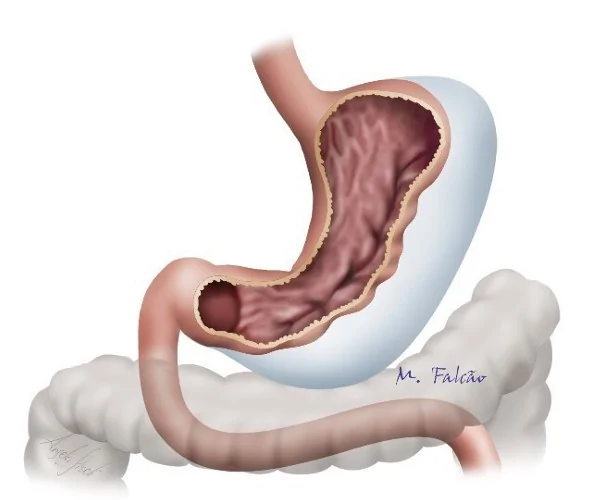
Posterior wall of the stomach showing the appearance of the procedure after suturing.

### Data collection

 Data collection was performed through the private service’s own platform. The main author tabulated a spreadsheet in Microsoft Excel^®^ with patient data obtained from medical records. 

### Epidemiological data

 Sex, age, body mass index (BMI) (kg/m^2^), weight (kg), excess weight loss (%EWL), and total weight loss (%TWL) were tabulated. The guidelines of the World Health Organization were followed when considering BMI to determine overweight (25–29.9 kg/m^2^), grade I obesity (30–34.9 kg/m^2^), grade II obesity (35–39.9 kg/m^2^), and grade III obesity (≥40 kg/m^2^). 

### Laboratory tests

 Laboratory test data corresponding to before surgery and to 1 month and 6 months after surgery during the follow-up period were added to the table. Thus, the results of total protein, albumin, calcium, zinc, vitamin B12, 25-hydroxyvitamin D, hemoglobin, glucose, glycated hemoglobin (HbA1c), ferritin, and iron were added to the spreadsheet. No tests beyond those routinely required at the research site were requested during patient follow-up. 

### Statistical analysis

 Statistical analysis was performed using SPSS version 25.0 (IBM SPSS, Chicago, IL). After testing the normality of quantitative variables (age, BMI, weight, %EWL, %TWL, and laboratory data) using the Kolmogorov-Smirnov test, the results were expressed as mean and standard deviation or median and interquartile range, depending on normality. Qualitative variables were expressed as percentages (sex). The ANOVA test with Bonferroni analysis was used to compare mean weight and BMI throughout the follow-up period. Comparisons before and after the procedure were performed using the paired t-test for variables expressed as means or the Mann-Whitney U test for variables expressed as medians. To correlate BMI with nutritional data, Pearson’s correlation test or Spearman’s test was used, depending on the normality of the variables. Correlations above 0.75 were considered strong, between 0.5 and 0.75 moderate, and less than 0.5 weak. Values of p<0.05 were considered statistically significant. 

## RESULTS

 Ninety-six patients were recruited for the study, of whom 69 were excluded according to the criteria presented in Figure 1. Thus, 27 patients were included in the study, with a mean age of 43.8 (±13.3) years. The majority (88.9%) of the sample was female, with a mean weight of 91.4±13.4 kg and a BMI of 35.31±8.7 kg/m^2^, as observed in Table 1. 

**Table 1:** Characteristics of patients before endoscopic gastroplasty.

Characteristic	EG (n=27)
Age,y (mean±SD)	43.8 (±13.3)
Female (%)	88.9%
BMI, Kg/m^2^ (mean±SD)	35.31±8.7
25–29.9 (n)	4
30.0–34.9 (n)	16
35.0–39.9 (n)	3
≥40 (n)	4
Weight, Kg (mean ±SD)	91.4±13.4

EG: endoscopic gastroplasty; SD: standard deviation; BMI: body mass index

 After 1 month following the procedure, there were no significant changes in laboratory parameters, with means/medians within normal values, indicating nutritional and metabolic stability during this period. At 6 months of follow-up, only data on vitamin B12, 25-hydroxyvitamin D, zinc, glucose, iron, and ferritin were recorded in the medical records, and no changes were observed, as shown in Table 2. 

**Table 2:** Statistics of laboratory variables throughout the follow-up period.

	Pre-EG	1 month	p-value	6 months	p-value
Hemoglobin (mean±SD)	12.5±0.9	12.8±0.7	0.10*	-	-
Ferritin (mean±SD)	109.1±70.1	130.70±76.1	0.39*	108.5±65.3	0.45*
Iron (mean±SD)	92.4±41.4	84.4±29.4	0.61*	85.2±36.1	0.52*
Calcium (median, IQR)	9.4 IQR 25–75	9.6 IQR 25–75	0.8**	-	-
Zinc (mean±SD)	89.8±26.6	88.7±14.2	0.3*	106±15.5	0.8*
Vitamin B12 (mean±SD)	424.1±211.2	486±159.6	0.62*	533±163.2	0.3*
25-OH-vitamin D (mean±SD)	33.7±11.9	37.5±5.8	0.92*	36.5±13.2	0.60*
Glucose (mean±SD)	95.5±13.6	83.3±8	0.06*	84.5±6	0.08*
HbA1C (mean±SD)	5.2±0.3	5.1±0.3	0.43*	-	-
Protein (median, IQR)	6.8 IQR 25–75	6.5 IQR 25–75	0.25**	-	-
Albumin (median, IQR)	4.5 IQR 25–75	4.6 IQR 25–75	0.54**	-	-

EG: endoscopic gastroplasty; SD: standard deviation; IQR: interquartile range; HbA1c: glycated hemoglobin; The paired t-test or Mann-Whitney U test was used to compare pre- and post-procedure values during follow-up;

* Paired t-test applied (sample represented by means);

** Mann-Whitney U test applied (sample represented by medians).

 Before the procedure, one patient presented with iron deficiency (Fe<30 ng/dL), two patients with calcium deficiency (<8.5 mg/dL), one patient with zinc deficiency (<70 μg/dL), two patients with vitamin B12 deficiency (<200 pg/dL), two patients with 25-hydroxyvitamin D deficiency (<20 ng/dL), and four patients with elevated fasting glucose (>99 mg/dL). After the procedure, no laboratory changes were observed among the 27 patients who completed follow-up. 

 No correlation was demonstrated between BMI and laboratory variables before the procedure, indicating that, in this small sample, BMI was not correlated with laboratory results, as shown in Table 3. 

**Table 3:** Correlation between body mass index and laboratory data prior to endoscopic gastroplasty.

Characteristic	Correlation	p-value
Hemoglobin	0.3	0.6*
Ferritin	0.4	0.07*
Iron	0.6	0.2*
Calcium	-0.1	0.1**
Zinc	0.2	0.06*
Vitamin B12	0.1	0.7*
25-OH-vitamin D	0.1	0.1*
Glucose	0.7	0.08*
HbA1c	-0.2	0.3*
Protein	0.6	0.05**
Albumin	0.2	0.1**

The Spearman test or Pearson correlation test was used depending on the normality of the laboratory variable, to correlate BMI with laboratory data; HbA1c: glycated hemoglobin;

* Pearson correlation test applied;

** Spearman test applied.

## DISCUSSION

 The present study found that the nutritional profile of this population remained stable after EG intervention in the short term (1–6 months). Although a few patients presented with deficiencies in iron, calcium, zinc, vitamin B12, and 25-hydroxyvitamin D before EG, there was no statistically significant impact in the short term. 

 These findings partially corroborate the literature. Ghoz et al.^12^ conducted a cohort study of 20 patients undergoing EG who were followed for 1 year and identified preexisting deficiencies in iron (18.2%) and ferritin (36.4%), but also reported additional deficiencies, such as anemia (22.2%), vitamin A (8.3%), vitamin E (18.2%), vitamin C (14.2%), magnesium (10%), and 25-hydroxyvitamin D (9%) postoperatively. Another study, also with 20 patients with a 2-year follow-up, showed low concentrations of 25-hydroxyvitamin D (80%), iron (40%), and vitamin C (5%) before treatment and a significant decrease in vitamin B12, B1, and B6 levels after EG^25^ . This divergence in the postoperative period can be explained both by the small sample size of the present sample and the studies analyzed and by the lack of data from the present sample. Furthermore, multivitamin/mineral supplementation for all patients was protocolized only by Ghoz et al.^12^ , but apparently was not sufficient to prevent nutritional deficiencies. 

 EG uses a restrictive mechanism for weight loss, mimicking laparoscopic vertical gastrectomy (SG)^11^ . Comparatively, this surgery presents more pronounced micronutrient alterations, such as hemoglobin deficiency (27%), vitamin B12 deficiency (4%), folic acid deficiency (10.2%), iron deficiency (32%), ferritin deficiency (11.2%), calcium deficiency (36%), zinc deficiency (12.5%), and 25-hydroxyvitamin D deficiency (9.7%)^6,13,18^. Little or no hypoalbuminemia has also been reported with this surgical technique^13^ . Although both are procedures that alter gastric volume, the lower degree of anatomical invasiveness of EG seems to preserve a greater degree of micronutrient absorption capacity. 

 ASMBS recommends nutritional screening for folic acid, iron, vitamin B12, D, A, E, K, and zinc. Furthermore, systematic replacement of vitamin B12 (300–500 mcg/day), folic acid (400–800 mcg/day), iron (18 mg/day), vitamin D (1,200–1,500 mg/day), zinc (8–11 mg/day), and copper (1 mg/day) is recommended in patients after bariatric surgery^1,8^. These guidelines are based on the high rates of deficiencies observed with techniques such as gastric bypass and SG. Despite the small sample size, the present study suggests that EG, by preserving intestinal transit, may require a more individualized approach regarding the need for supplementation. 

 Long-term follow-up data demonstrate a significant effect of EG on DM2 control of and glycemic homeostasis. This is associated with weight reduction and reduced insulin resistance. Hormonal influences and changes in dietary habits should also be considered^4,15,16,23^. Dayyeh et al.^7^ reported DM2 control in 92% of the 209 patients undergoing EG analyzed in the MERIT TRIAL. In this small sample, no significant metabolic changes were observed in DM2 control. 

 This study has some limitations. Among them, the limited number of patients compromises a robust analysis of the data. Loss to follow-up over the 6-month of monitoring period leads to attrition bias, since it is not possible to determine in the long term whether nutritional levels fluctuate or remain within normal parameters. The lack of complete data itself can lead to information bias. The lack of a control group of patients with obesity who did not undergo EG limits the ability to associate the endoscopic procedure with laboratory data. In addition, the study was conducted at a single center, which may lead to selection bias. 

 Thus, the present research adds data on a topic that remains relatively underexplored: the influence of EG on the nutritional profile of patients with obesity. Prospective studies with larger samples and prolonged follow-up are needed to confirm these findings and establish clear guidelines regarding the need for vitamin supplementation after EG. 

## CONCLUSIONS

 In the sample studied, the nutritional profile remained stable before and after treatment with EG in the short term (up to 6 months). Sustained weight loss was observed throughout the follow-up period, and BMI and laboratory data before the procedure were not correlated. However, due to the reduced statistical power of this sample and the incompleteness of the data, the implications of EG for potential nutritional deficiencies in the management of obesity remain uncertain. 

## Data Availability

The datasets generated and/or analyzed during the current study are available from the corresponding author upon reasonable request.
